# Supplementary material for: Integrated analysis of the aqueous humor microbiome and lens capsule transcriptome in high myopia cataract: a pilot study
Source: Front Med (Lausanne). 2026 Jun 16;13:1845205. doi: 10.3389/fmed.2026.1845205 (PMC13314463; doi:10.3389/fmed.2026.1845205)
Supplement: Supplementary file 3 [file Data_Sheet_1.zip › 3.Beta_Diversity/PCoA/C372089/PCoA_bray_curtis_3D_nolabel.pdf]

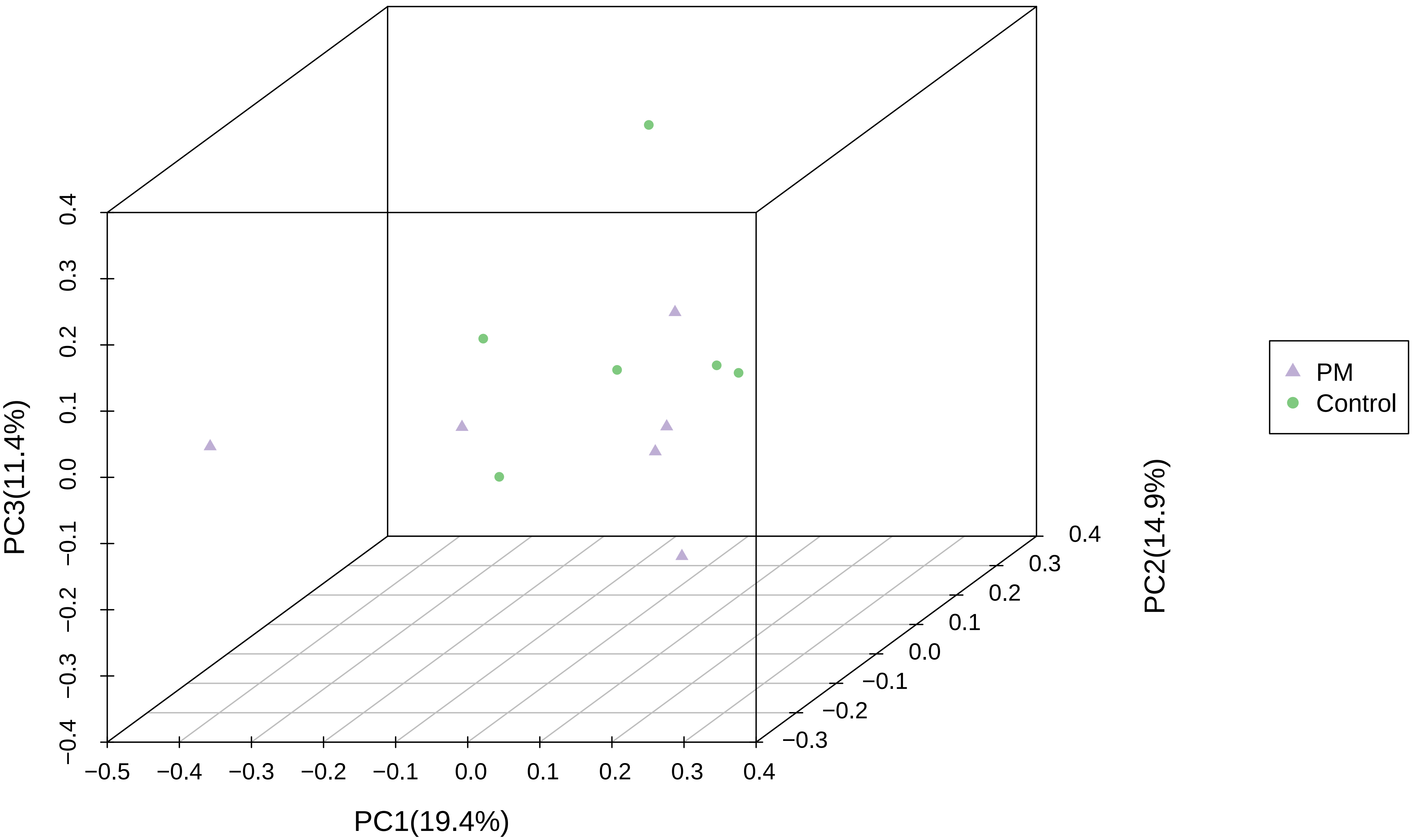

PM
Control
PC1(19.4%)
PC2(14.9%)
PC3(11.4%)
−0.5
−0.4
−0.3
−0.2
−0.1
0.0
0.1
0.2
0.3
0.4
